# FACS-based genome-wide CRISPR screening platform identifies modulators of CD47

**DOI:** 10.3389/fimmu.2025.1684539

**Published:** 2026-01-12

**Authors:** Ling Yin, Wei He, Yifan Wang, Huimin Zhang, Min Huang, Yuelong Yan, Siting Li, Xu Feng, Francisco Saenz, Jie Zhang, Dandan Zhu, Chang Yang, Tiantian Ma, Jialing Fu, Junjie Chen

**Affiliations:** 1 Department of Experimental Radiation Oncology, The University of Texas MD Anderson Cancer Center, Houston, TX, United States; 2 Department of Epigenetics & Molecular Carcinogenesis, The University of Texas MD Anderson Cancer Center, Houston, TX, United States; 3 Department of Radiation Oncology, The University of Texas MD Anderson Cancer Center, Houston, TX, United States

**Keywords:** FACS, CRISPR/Cas9 screen, CD47, macrophage, DNAJC13

## Abstract

**Background:**

CD47 is a key innate immune checkpoint that enables tumor cells to evade macrophage-mediated clearance.

**Methods/Results:**

To systematically identify genetic regulators of CD47 surface expression, we performed FACS-based genome-wide CRISPR screens in three murine cancer cell lines B16 (melanoma), MC38 (colon adenocarcinoma), and EMT6 (breast carcinoma).

**Results:**

Comparative analysis of cells with high or low CD47 surface expression using DrugZ revealed CD47 itself as the top hit, validating the screens. Notably, DNAJC13 emerged as a consistent and robust regulator of CD47 expression across all three cell lines. Functional validation using DNAJC13-knockout cells confirmed a significant reduction in CD47 surface levels. Furthermore, in co-culture assays with macrophages, DNAJC13-deficient tumor cells exhibited increased susceptibility to phagocytosis, supporting a functional role for DNAJC13 in innate immune evasion. Finally, we verify that DNAJC13-knockout decrease tumor burden when treated with CD47 blockade.

**Conclusions:**

Overall, this study highlights a previously unrecognized regulator of CD47 and demonstrates the utility of high-throughput FACS-based CRISPR screening to uncover modulators of key immune checkpoint pathways.

## Introduction

High-throughput CRISPR-Cas9 screening has emerged as a transformative tool for systematic functional genomics, enabling the unbiased identification of genes that regulate diverse cellular phenotypes (1–4). Traditional pooled CRISPR screens often rely on cell survival, proliferation, or reporter-based readouts to infer gene function. While powerful, these approaches are limited in their ability to interrogate phenotypes that are not easily linked to cell fitness or transcriptional activity.

Fluorescence-activated cell sorting (FACS)-based CRISPR screens overcome these limitations by allowing direct measurement and enrichment of cells based on surface protein expression, signaling states, or other cellular features detectable by flow cytometry (5, 6). This strategy is particularly well-suited for immune-related studies, where the surface expression of immune checkpoints, ligands, or modulators is central to tumor-immune interactions. By physically separating high- and low-expressing populations, FACS-based screens enable quantitative analysis of gene knockouts that alter protein abundance at the single-cell level, a key advantage for dissecting the regulation of immunologically relevant molecules.

CD47 is a transmembrane protein broadly expressed on normal and malignant cells, often referred to as a “don’t eat me” signal due to its interaction with signal regulatory protein alpha (SIRPα) on macrophages (7, 8). This interaction inhibits phagocytosis, allowing tumor cells to evade innate immune surveillance. Many cancers, including hematologic malignancies and solid tumors, overexpress CD47 to escape immune-mediated clearance. As a result, CD47 has emerged as a promising immunotherapeutic target. Preclinical studies have shown that blocking the CD47–SIRPα axis can restore macrophage-mediated phagocytosis and enhance antigen presentation, thereby promoting both innate and adaptive antitumor immunity. Several CD47-targeting agents, including monoclonal antibodies and SIRPα-Fc fusion proteins, have entered clinical trials. Notably, magrolimab (an anti-CD47 antibody) has shown encouraging activity, particularly in combination with azacitidine in patients with myelodysplastic syndromes (MDS) (9) and acute myeloid leukemia (AML) (10), and is now being evaluated in a range of cancers, both hematologic and solid. Although CD47 is a critical mediator of immune evasion and a target of active immunotherapeutic development (11, 12), the genetic mechanisms that govern its surface expression remain incompletely defined.

In this study, we applied a FACS-based, genome-wide CRISPR knockout screening platform to explore regulators of CD47. We performed whole-genome CRISPR screens in three murine cancer cell lines, B16 (melanoma), MC38 (colon adenocarcinoma), and EMT6 (breast carcinoma). We used FACS to isolate the top and bottom 30% of CD47-expressing cells. This strategy allowed us to identify both positive and negative regulators of CD47 surface abundance. Our analysis revealed a conserved role for DNAJC13, a gene not previously associated with immune regulation, in promoting CD47 expression. Functional validation demonstrated that DNAJC13 knockout reduces CD47 levels and enhances macrophage-mediated phagocytosis, uncovering a novel regulator of innate immune escape.

## Results

### Whole-genome CRISPR screens of CD47 expression in three mouse tumor cell lines

**Figure 1:**
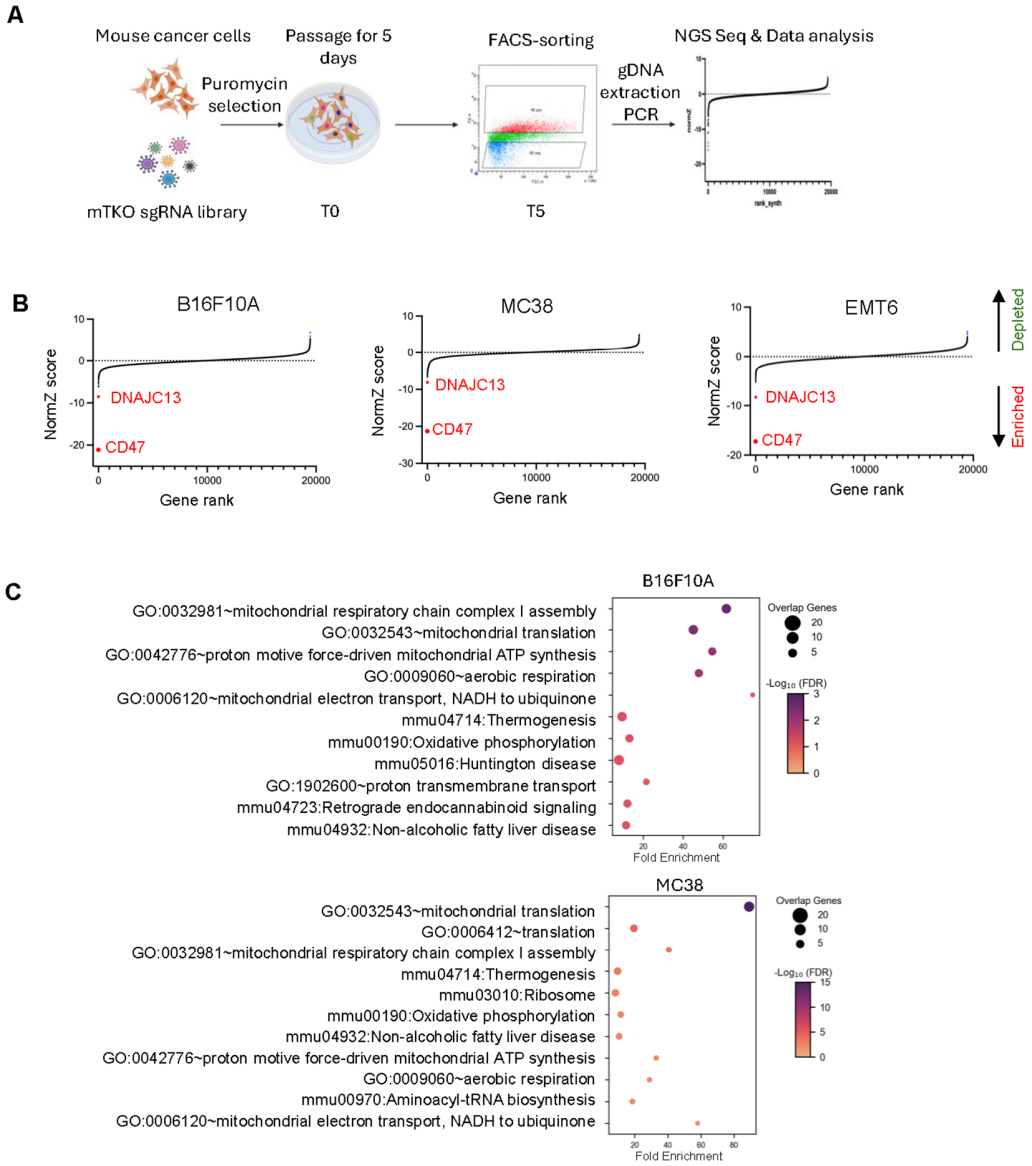
Genome-wide CRISPR-Cas9 screen identifies genetic regulators of CD47 expression in murine cancer cell lines. **(A)** Schematic of the FACS-based CRISPR screening pipeline. Mouse cancer cells (B16F10A, MC38, and EMT6) were transduced with the mTKO genome-wide CRISPR-Cas9 library, followed by puromycin selection and passaging for 5 days to ensure stable sgRNA integration. Cells were stained with fluorophore-conjugated anti-CD47 antibodies and sorted by FACS into the top 30% (CD47^high^) and bottom 30% (CD47^low^) populations. Genomic DNA was extracted, and sgRNA abundance was determined by NGS and analyzed using DrugZ to calculate NormZ scores. **(B)** Ranked NormZ scores of sgRNAs in B16F10A, MC38, and EMT6 cells. Negative NormZ scores indicate positive regulators of CD47 (gene knockouts reduce CD47 expression), while positive NormZ scores indicate negative regulators (gene knockouts increase CD47 expression). CD47 itself ranked as the top positive regulator in all three cell lines, validating the screen’s robustness. **(C)** GO enrichment analysis of significant negative regulators of CD47 expression (|NormZ| > 3) in B16F10A and MC38 cells. Negative regulators were significantly enriched in pathways related to mitochondrial signaling, including oxidative phosphorylation, mitochondrial translation, and respiratory chain complex assembly.

Given the critical role of the CD47–SIRPα axis in protecting tumor cells from macrophage-mediated phagocytosis, we sought to systematically identify tumor-intrinsic genetic regulators of CD47 expression. To this end, we established a FACS-based, genome-wide CRISPR knockout screening pipeline targeting CD47 in three murine cancer cell lines (MC38, B16, and EMT6). Tumor cells were transduced with the mouse whole-genome mTKO CRISPR-Cas9 library (13), an all-in-one lentiviral vector–based library optimized for high sgRNA editing efficiency. After puromycin selection, we then cultured single-guide RNA (sgRNA)-infected cells for 5 days. On day 5 of the screening, cells were stained with fluorophore-conjugated anti-CD47 antibodies and sorted by flow cytometry based on surface CD47 expression levels. Specifically, the top 30% (CD47^high^) and bottom 30% (CD47^low^) populations were collected for downstream analysis. Genomic DNA was extracted from each sorted population, and sgRNA abundance was quantified by next-generation sequencing (NGS) (**Figure 1A**).

The distribution of read counts in different conditions is shown in **Supplementary Figure S1A**, the similar median read counts (green lines), interquartile ranges (boxes), and overall distribution patterns across conditions suggest uniform sgRNA representation and consistent library performance, indicating that the genome-wide CRISPR screen was robust and technically reliable. DrugZ (14) enrichment analysis was then performed to compare sgRNA representation between the CD47^low^ and CD47^high^ populations. In this analysis, negative NormZ values indicate positive regulators of CD47 (i.e., gene knockouts that reduced CD47 expression), whereas positive NormZ values indicate negative regulators of CD47 (i.e., gene knockouts that increased CD47 expression). This strategy enabled the unbiased identification of candidate genetic regulators influencing CD47-mediated phagocytosis (**Supplementary Tables S1**). As expected, CD47 itself ranked as the top positive regulator across all three cell lines (**Figure 1B**), validating the robustness and reliability of the screening pipeline.

Applying stringent selection criteria (|NormZ| > 3), we identified 196, 94, and 51 positive regulators and 41, 75, and 34 negative regulators of CD47 expression in MC38, B16, and EMT6 cells, respectively. Among these were several previously reported CD47 regulators, including STAT3, a transcription factor known to directly bind the CD47 promoter and upregulate CD47 in tumors upon cytokine activation (15); HIF1A/HIF2A, which enhance CD47 expression under hypoxic conditions (16); and RUNX1/RUNX2, which have been implicated in modulating immune-related transcriptional programs, including CD47, in hematopoietic and solid tumors (17). The recovery of these known regulators supports the feasibility of our screens to identify both established and novel regulators of CD47 expression. To gain functional insights into the underlying biology, we performed Gene Ontology (GO) enrichment analysis on the identified regulators using DAVID web server (**Supplementary Tables S2**) (18). While no significant GO terms were enriched in the EMT6 dataset (**Supplementary Figure S1C**), negative regulators identified in MC38 and B16 cells were significantly enriched in pathways associated with mitochondrial signaling (**Figure 1C**). Mitochondrial function is known to play a crucial role in macrophage phagocytosis by regulating energy production, ROS signaling, and innate immune activation (19–21). The identification of these genes suggests that tumor-intrinsic mitochondrial pathways may influence macrophage-mediated immune surveillance.

### Loss of DNAJC13 decreases CD47 expression

**Figure 2:**
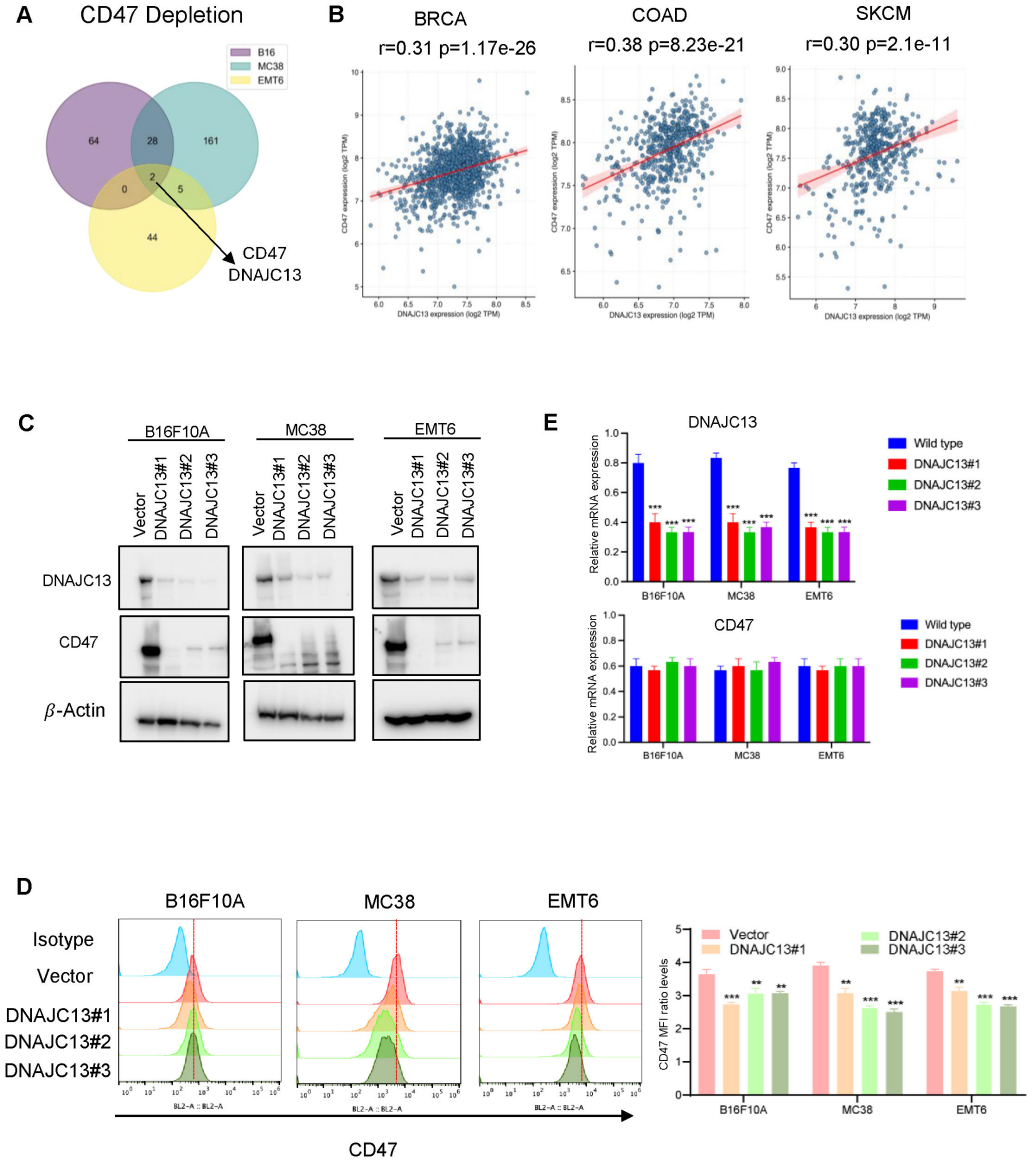
DNAJC13 is a conserved positive regulator of CD47 expression identified across multiple cancer cell lines. **(A)** Venn diagram of significant positive regulators of CD47 expression identified in the genome-wide CRISPR screen. CD47 and DNAJC13 were the only two genes consistently identified as significant positive regulators (|NormZ| > 3) across all three cell lines (B16F10A, MC38, and EMT6). **(B)** Correlation of DNAJC13 and CD47 expression in human cancers. Scatter plots generated from TCGA datasets via GEPIA2 show a positive correlation between DNAJC13 and CD47 expression in breast cancer (BRCA), colon adenocarcinoma (COAD), and skin cutaneous melanoma (SKCM). Pearson correlation coefficients (r) and p-values are indicated. **(C)** Western blot validation of DNAJC13 regulation of CD47 expression. CRISPR-Cas9–mediated DNAJC13 knockout (three independent sgRNAs: DNAJC13#1, #2, #3) markedly reduced CD47 protein levels compared with vector control in B16F10A, MC38, and EMT6 cells. GAPDH was used as a loading control. **(D)** Flow cytometry analysis of surface CD47 expression. Representative FACS histograms show reduced surface CD47 expression in DNAJC13 KO cells (green, yellow, and orange peaks; three independent sgRNAs) compared with vector controls (red) in B16F10A, MC38, and EMT6 cells. Isotype control is shown in blue. Quantification of Mean fluorescence intensity (MFI) is shown on the right.

Among all positive regulators identified in the FACS-based CRISPR screen, two genes consistently emerged as top hits across all three cell lines: CD47 and DNAJC13 (**Figure 2A**). Indeed, DNAJC13 consistently appeared as the second highest-ranked gene in all three cell lines, suggesting a previously unrecognized role in regulating CD47 expression or surface presentation. To investigate the potential relationship between DNAJC13 and CD47, we first examined their expression patterns in public datasets. Analysis of the Cancer Cell Line Encyclopedia (CCLE) (22) revealed a positive correlation between DNAJC13 and CD47 expression across multiple cancer cell lines, including breast cancer, melanoma, and colon cancer. Consistent with this observation, analysis of TCGA datasets using the GEPIA web tool (23) demonstrated that DNAJC13 expression is strongly and positively correlated with CD47 in several cancer types, particularly in breast cancer, melanoma, and colon cancer (**Figure 2B**). These findings suggest a potential regulatory relationship between DNAJC13 and CD47 in multiple cancer types.

To functionally validate this correlation, we hypothesized that knockdown of DNAJC13 would reduce CD47 expression. We established DNAJC13 knockout (KO) cell lines in three different murine cancer models (EMT6, MC38, and B16F10A) using CRISPR-Cas9–mediated gene editing. Western blotting confirmed efficient depletion of DNAJC13 protein in all three KO cell lines (**Figure 2C**). Notably, CD47 protein levels were markedly reduced in DNAJC13-deficient cells, suggesting a positive regulatory relationship between DNAJC13 and CD47. Flow cytometry analysis further revealed a consistent decrease in CD47 surface expression across all three models (**Figure 2D**).

To begin exploring the underlying mechanism, we asked whether DNAJC13 regulates CD47 at the transcriptional or post-transcriptional level. Although CD47 protein levels were clearly reduced, we did not observe a significant change in CD47 mRNA levels in DNAJC13 KO cells (**Figure 2E**), suggesting that the effect is likely post-transcriptional. Given the known role of DNAJC13 in endosomal trafficking and receptor recycling, we hypothesize that DNAJC13 may stabilize CD47 at the plasma membrane or prevent its degradation through vesicular trafficking pathways. Further mechanistic studies will be needed to directly test these possibilities.

### DNAJC13 depletion increase macrophage phagocytosis by downregulating CD47

**Figure 3:**
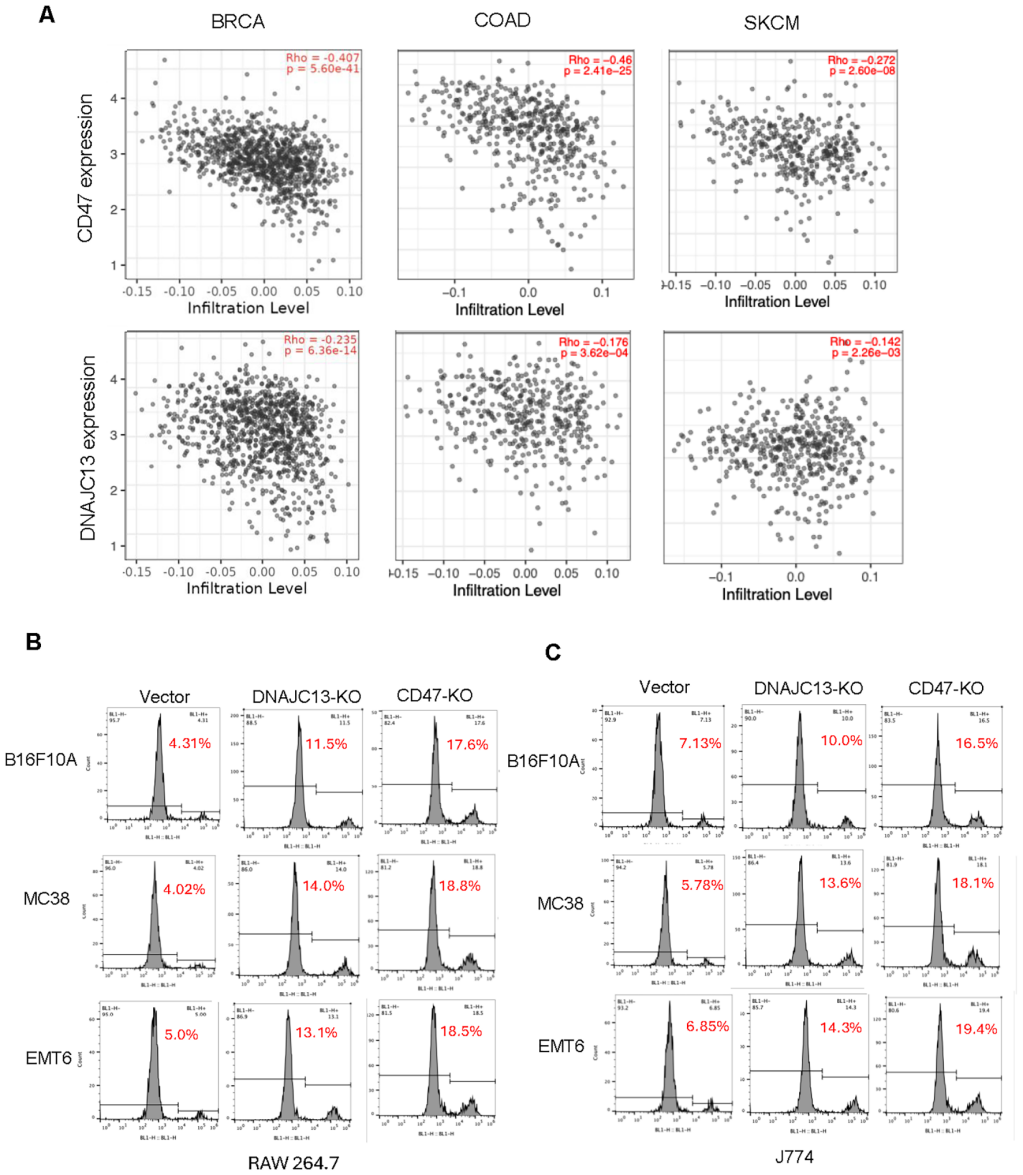
DNAJC13 negatively correlates with macrophage infiltration and regulates macrophage-mediated phagocytosis. **(A)** Correlation between gene expression and macrophage infiltration in human cancers. Scatter plots generated using TIMER2 show negative correlations between CD47 expression (top row) or DNAJC13 expression (bottom row) and macrophage infiltration levels in breast cancer (BRCA), colon adenocarcinoma (COAD), and skin cutaneous melanoma (SKCM). Spearman’s correlation coefficients (Rho) and p-values are indicated. **(B, C)** DNAJC13 loss enhances macrophage phagocytosis *in vitro*. Flow cytometry analysis of macrophage-mediated phagocytosis in co-culture assays. DNAJC13 knockout (KO) or CD47 KO cancer cells (B16F10A, MC38, EMT6) were co-cultured with **(B)** RAW 264.7 or **(C)** J774 macrophages for the indicated time. Representative FACS plots show increased phagocytosis (higher engulfment rate) in DNAJC13-KO and CD47-KO groups compared with vector controls.

Given the established role of the CD47–SIRPα axis in enabling tumor immune evasion by suppressing macrophage phagocytosis, we next investigated whether DNAJC13 influences macrophage infiltration in human cancers. Using the TIMER2 web application (24), we systematically analyzed correlations between gene expression and immune cell infiltration across multiple TCGA cancer types. As expected, CD47 expression was strongly and negatively correlated with macrophage infiltration in several cancer types, including breast cancer, melanoma, and colon cancer, consistent with its well-known function as a “don’t eat me” signal that inhibits macrophage-mediated phagocytosis. Notably, DNAJC13 expression displayed a similar negative correlation with macrophage infiltration (**Figure 3A**), suggesting that DNAJC13 may contribute to tumor immune evasion by maintaining high CD47 expression levels, in agreement with our CRISPR screening results that identified DNAJC13 as a positive regulator of CD47 surface expression.

To experimentally validate the functional consequence of DNAJC13 loss on tumor–macrophage interaction, we performed macrophage co-culture assays to directly assess phagocytosis efficiency. We first established CD47 knockout (KO) cancer cell lines using CRISPR-Cas9 and confirmed effective reduction of both CD47 mRNA and protein levels (**Supplementary Figures S3A, B**). These CD47 KO cells, along with DNAJC13 KO and parental control cells, were co-cultured with two independent murine macrophage lines—RAW 264.7 and J774—under standardized conditions. As expected, CD47-deficient tumor cells were robustly phagocytosed, reflecting the loss of the “don’t eat me” signal. Notably, DNAJC13 knockout cells also exhibited significantly increased phagocytosis compared to parental controls, though to a lesser extent than CD47 KO cells (**Figures 3B, C**, **Supplementary Figure S3C**). This intermediate phenotype supports the hypothesis that DNAJC13 contributes to immune evasion by regulating CD47 surface expression, and that its loss only partially phenocopies CD47 deletion. The consistent enhancement of phagocytosis across both macrophage cell lines suggests the effect is not macrophage-type specific, but rather intrinsic to the tumor cells. Together, these results demonstrate that loss of DNAJC13 sensitizes tumor cells to macrophage-mediated phagocytosis, likely by disrupting CD47-dependent inhibitory signaling, and reinforces the functional relevance of DNAJC13 as a modulator of innate immune evasion.

### DNAJC13 depletion increase macrophage phagocytosis *in vivo*

**Figure 4:**
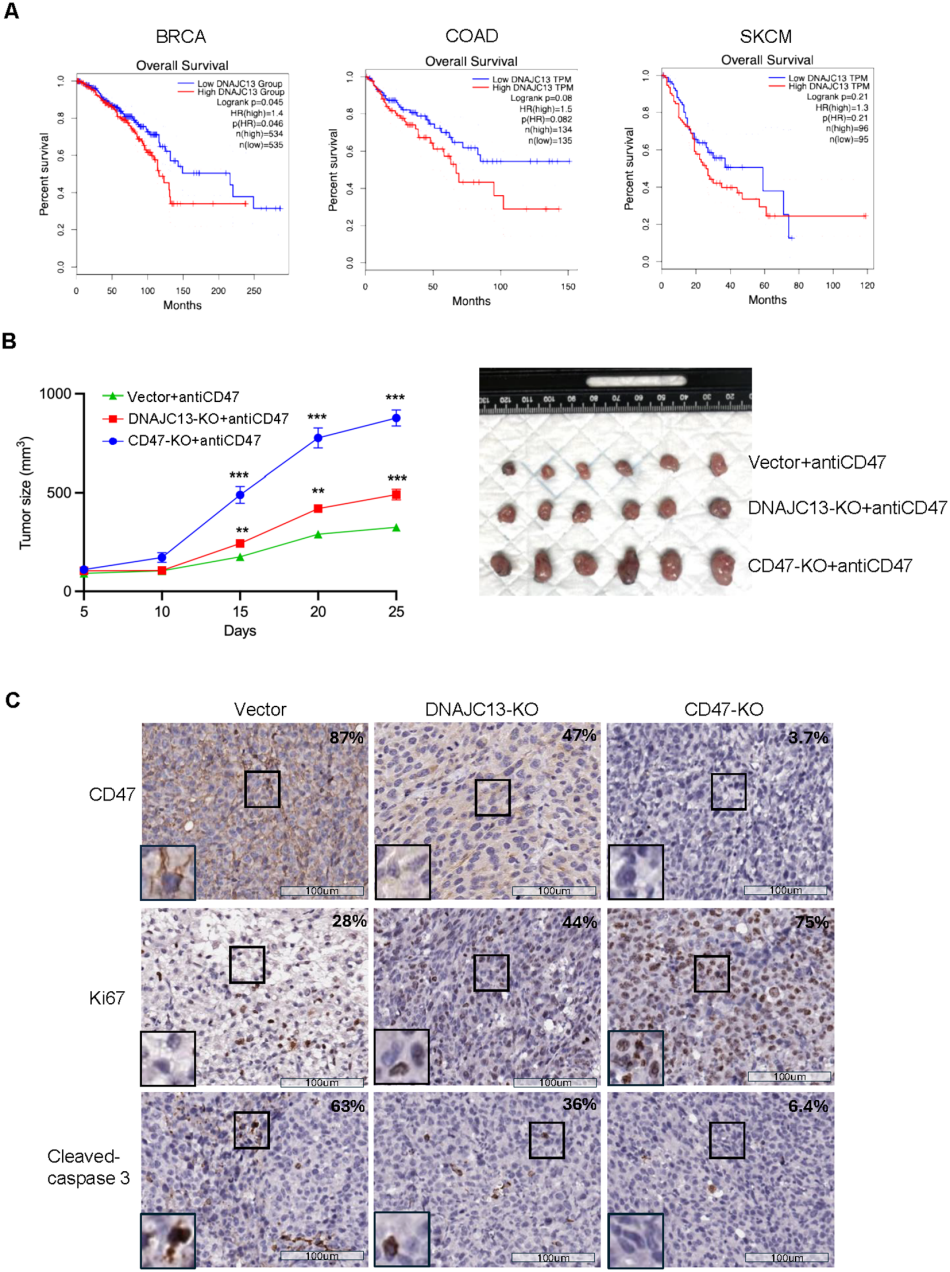
Clinical relevance of DNAJC13 expression and its impact on tumor growth *in vivo*. **(A)** High DNAJC13 expression is associated with improved overall survival in human cancers. Kaplan–Meier survival curves generated from TCGA datasets via GEPIA2 show overall survival (OS) in patients with breast cancer (BRCA), colon adenocarcinoma (COAD), and skin cutaneous melanoma (SKCM), stratified by high (red) versus low (blue) DNAJC13 expression. Log-rank p-values, hazard ratios (HR), and sample sizes (n) are indicated. **(B)** DNAJC13 loss reduces tumor growth *in vivo*. (Left) Tumor growth curves of B16F10A tumors in immune-competent mice treated with anti-CD47 monoclonal antibody. DNAJC13-KO tumors exhibited significantly reduced growth compared with vector controls, and tumor growth in DNAJC13-KO mice was comparable to that in the CD47-KO group, suggesting that DNAJC13 regulates tumor growth primarily through CD47. Data are presented as mean ± SEM; statistical significance was determined by two-way ANOVA (**p < 0.01, ***p < 0.001). (Right) Representative images of excised tumors from each group at the study endpoint. **(C)** CD47, Ki-67 (proliferation) and cleaved caspase-3 (apoptosis) staining were conducted in tumors shown in **(B)**.

Given our findings that DNAJC13 knockdown enhances macrophage-mediated phagocytosis *in vitro*, we hypothesized that DNAJC13 expression may influence patient prognosis by modulating anti-tumor immune responses *in vivo*. To test this hypothesis, we analyzed TCGA clinical datasets (25) to evaluate the association between DNAJC13 expression and patient outcomes. Interestingly, high DNAJC13 expression was significantly associated with poor overall survival in breast cancer (BRCA, p=0.045), and colon adenocarcinoma (COAD, p=0.008), while the association is not statistically significant in melanoma (SKCM, p = 0.21) (**Figure 4A**). This observation suggests that DNAJC13 plays an important role in tumor biology, potentially by regulating CD47-mediated macrophage phagocytosis *in vivo*.

To further validate the functional role of DNAJC13 in regulating tumor immune evasion, we performed *in vivo* studies using immune-competent mice. Subcutaneous tumors were established using the three cell lines, and tumor growth was monitored in mice bearing vector control, DNAJC13 knockout, and CD47 knockout tumors (shown in **Supplementary Figure S3D**). In the absence of treatment, tumor growth was comparable among all three groups, indicating that neither CD47 nor DNAJC13 knockout alone significantly suppresses tumor progression *in vivo*.

We next tested whether DNAJC13 knockout could enhance the therapeutic response to CD47 blockade. In a parallel cohort of mice, we treated tumors with an anti-CD47 monoclonal antibody following a standard dosing schedule. These experiments were performed concurrently under identical conditions, allowing direct comparison. As shown in **Figure 4B**, tumors lacking DNAJC13 exhibited enhanced sensitivity to CD47 blockade, with significantly reduced tumor volume compared to control tumors treated with antibody. Interestingly, anti-CD47 treatment in CD47 KO tumors had no additional effect, as expected. Notably, while DNAJC13 KO and CD47 KO tumors grew similarly without treatment (**Supplementary Figure S3D**), they responded differently to anti-CD47 therapy: DNAJC13 KO tumors responded significantly, whereas CD47 KO tumors did not, confirming the on-target effect of CD47 inhibition. Although *in vivo* phagocytosis was not directly measured, the enhanced response of DNAJC13-deficient tumors to CD47 blockade supports the conclusion that DNAJC13 loss increases macrophage-mediated antitumor activity.

To further characterize the biological effects of DNAJC13 loss in tumors, we performed immunohistochemistry (IHC) on harvested tumor tissues to evaluate cell proliferation and apoptosis. Tumors derived from DNAJC13 KO cells exhibited significantly decreased Ki67 expression and increased cleaved caspase-3 levels, indicating reduced proliferation and enhanced apoptosis (**Figure 4C**). Interestingly, these changes were not observed in vector control tumors, suggesting our conclusion that DNAJC13 promotes immune evasion through CD47-dependent mechanisms, and that its loss can sensitize tumors to CD47 blockade.

## Discussion

In this study, we used a FACS-based, genome-wide CRISPR-Cas9 knockout screening approach to identify genetic regulators of CD47 surface expression in murine cancer cells. CD47 is a critical innate immune checkpoint that enables tumor cells to evade macrophage-mediated phagocytosis, and its overexpression is a well-established mechanism of immune escape across multiple cancer types. While CD47-targeting agents are under active clinical investigation, the regulatory networks governing CD47 expression remain poorly defined. Our screen provides new insight into the upstream control of CD47 and identifies DNAJC13 as a previously unrecognized and conserved positive regulator of its surface expression.

DNAJC13 emerged as the top hit across all three murine tumor models (B16, MC38, EMT6), suggesting a robust and lineage-independent role in modulating CD47 levels. Functional validation confirmed that DNAJC13 knockout reduces CD47 surface expression and enhances macrophage-mediated phagocytosis *in vitro*. Moreover, *in vivo* studies demonstrated that DNAJC13-deficient tumors were more sensitive to anti-CD47 antibody therapy, resulting in reduced tumor growth and increased immune activation, as reflected by decreased Ki-67 and elevated cleaved caspase-3 staining. Together, these findings indicate that DNAJC13 contributes to tumor immune evasion through regulation of CD47 surface abundance.

DNAJC13 is a member of the DNAJ/HSP40 family and is known to be involved in endosomal trafficking and protein recycling, including receptor transport between endosomes and the plasma membrane. Its role in the immune context has not been previously characterized. We speculate that DNAJC13 may facilitate recycling or stabilization of CD47 at the cell surface, thereby maintaining its expression under homeostatic conditions. Loss of DNAJC13 may lead to CD47 internalization or degradation, rendering tumor cells more susceptible to innate immune clearance. Further mechanistic studies will be required to elucidate whether DNAJC13 acts through vesicular trafficking, post-translational modifications, or other processes affecting CD47 localization.

We also observed a correlation between DNAJC13 expression and macrophage infiltration in human breast cancer, suggesting a clinically relevant association and a potential role in shaping the tumor immune microenvironment. Consistent with our functional results, TCGA survival analysis indicated that high DNAJC13 expression correlates with poor overall survival in BRCA and COAD, while no significant association was observed in SKCM. These findings suggest that DNAJC13 may contribute to tumor progression and immune evasion in a context-dependent manner. DNAJC13 is a multifunctional trafficking regulator involved in the recycling and localization of various membrane proteins, including CD47. The context-dependent prognostic impact of DNAJC13 may reflect its diverse cellular functions and the complexity of tumor microenvironments across different cancers. Further investigation using larger and more homogeneous clinical cohorts will be necessary to clarify the prognostic and mechanistic roles of DNAJC13 in distinct tumor types.

Interestingly, despite the well-established role of CD47 in suppressing innate immune clearance, we did not observe significant tumor growth delay in mice bearing CD47- or DNAJC13-deficient tumors in the absence of therapeutic intervention. This may reflect the fact that CD47 blockade requires active engagement of macrophages and Fc receptor–mediated antibody activity to promote efficient phagocytosis. Several studies have shown that the efficacy of anti-CD47 therapies depends not only on disrupting the CD47–SIRPα interaction but also on the opsonizing and effector functions of the antibody Fc domain to trigger phagocytosis via macrophage Fcγ receptors (26–28). Therefore, in the absence of exogenous antibody, CD47 loss alone may be insufficient to overcome the phagocytic threshold *in vivo*. Similarly, while DNAJC13 knockout reduces CD47 expression, this reduction may not be sufficient to activate macrophage-mediated clearance without additional immune stimulation. In contrast, combining DNAJC13 knockout with anti-CD47 therapy significantly impaired tumor growth, suggesting that partial loss of CD47 primes tumors to respond more robustly to therapeutic blockade, possibly by enhancing the sensitivity of tumor cells to Fc-mediated effector mechanisms. These findings highlight the therapeutic potential of combining CD47 expression modulators with immune checkpoint–targeted antibodies and underscore the context-dependent nature of antitumor immune activation.

Our study has several important implications. First, it validates the power of FACS-based CRISPR screening as a platform for identifying regulators of cell surface immune checkpoint proteins. This method can be extended to study other immunoregulatory molecules in various tumor types. Second, it positions DNAJC13 as a potential therapeutic sensitizer. Its inhibition may enhance the efficacy of CD47-targeted therapies by reducing CD47 expression and increasing macrophage activity. Finally, our findings add to the growing appreciation of intracellular regulators of immune checkpoint biology, which may offer new intervention points beyond extracellular blockade.

Limitations of this study include the lack of mechanistic dissection of how DNAJC13 regulates CD47, and the focus on murine models. Given its known role in endosomal trafficking and receptor recycling, DNAJC13 may regulate CD47 surface expression by modulating its intracellular trafficking or stability. Future studies using biochemical and imaging approaches will be required to delineate the precise molecular interaction between DNAJC13 and CD47. Additional experiments in human cancer cells and investigation of direct molecular interactions between DNAJC13 and CD47 trafficking machinery would strengthen the translational potential of these findings. Although our findings were reproduced using three independent DNAJC13-targeting sgRNAs, a rescue experiment restoring DNAJC13 expression would further validate the causal relationship between DNAJC13 loss and reduced CD47 levels. Future work will incorporate such complementation assays to definitively confirm this mechanism. In addition, further studies with detailed immune profiling will help delineate how DNAJC13 loss modulates macrophage subsets and other immune cell populations in the tumor microenvironment.

In conclusion, we identify DNAJC13 as a conserved regulator of CD47 surface expression and innate immune evasion. Our results support further exploration of DNAJC13 as a therapeutic target or biomarker in CD47-directed immunotherapy and highlight the utility of FACS-coupled CRISPR screens to uncover modulators of immune checkpoint control.

## Materials and methods

### Cell lines

Mouse melanoma cell line B16F10A, mouse colon cancer cell line MC38, mouse breast cancer cell line EMT6, two mouse macrophage cells J774A-1, RAW264.7 are purchased from the American Type Culture Collection (ATCC). B16F10A, EMT6, J774A-1, RAW264.7 are cultured in Dulbecco’s modified Eagle’s medium (DMEM) supplemented with 10% fetal calf serum (Sigma). EMT6 cells are grown in Waymouth’s MB 752/1 Medium with 2mM L-glutamine and 15% fetal bovine serum. All the cell lines were tested to verify that they were free of mycoplasma contamination.

### FACS-based CRIPSR/Cas9 screening

The Mouse Toronto KnockOut (mTKO) CRISPR Library contains 94,528 gRNAs targeting 19,463 protein-coding genes (4 gRNAs/gene) and 418 EGFP-, LacZ-, and luciferase-targeted control gRNAs (29). This library is a gift from Dr Traver Hart (MD Anderson Cancer Center). The library generation and virus preparation were described in a previous paper (30). The screen was conducted as described. Briefly, the three mouse cancer cells were infected with the mTKO library lentivirus at an MOI of ~0.25 to keep every gRNA representation in at least ~250 cell. Twenty-four hours later, cells were cultured with fresh medium containing puromycin (2 μg/mL) for selection. After selection, cells were cultured and passaged for about 200~fold coverage. At day 5, 100 million cells per group collected in a 15-ml tube filled with buffer on a rotating mixer for immunostaining. Next, cells were incubated with indicated CD47 antibody (1:500 diluted in 3% bovine serum albumin) at room temperature 15mins and then wash with PBS three times. Finally, cells were resuspended in PBS for flow cytometry selection (the top 30% with the highest signals and bottom 30% with the lowest signals cell populations, respectively). Cells were collected for genomic DNA extraction (Qiagen Kit). PCR reaction was performed to amplify gRNAs inserts via primers harboring Illumina TruSeq adapters with i5 and i7 barcodes in a previous paper. The resulting libraries were sequenced using an Illumina HiSeq 2500 system. Model-based Analysis of Genome-wide CRISPR/Cas9 Knockout (MAGeCK) (31) and drug-Z (32) analysis were conducted to calculate the difference in gRNA enrichment between top 30% and bottom 30%.

### Generation of knock out cells

DNAJC13 and CD47-KO cells were generated by using pLentiCRISPRv2 plasmids. gRNA against target genes was designed by Synthego. Cells were transfected and selected with 2 mg/ml puromycin for 48 hours after transfection. Then the pool cells are collected for further verification by immunoblotting. gRNAs sequences used for the generation of knock out cells in this study were listed below:

DNAJC13-KO1: sgRNA: ACACAACCAAGCACTCATGG.

DNAJC13-KO2: sgRNA: CAGAACAGAGCTGCTTACGG.

DNAJC13-KO3: sgRNA: GAGCGATGCAAAGAAACCTG.

CD47-KO1: sgRNA: GGAGATGTGGCCCTTGGCGG.

CD47-KO2: sgRNA: AATGGATAAGCGCGATGCCA.

### Western blot analysis

Cells were lysed in 1 x SDS gel-loading buffer (50 mM tris-HCl (pH 6.8), 2% SDS, 10% glycerol, 4% beta-mercaptoethanol, and 0.025% bromophenol blue) and boiled 10 min for further analysis. Samples were separated by sodium dodecyl sulfate polyacrylamide gel electrophoresis and analyzed by immunoblotting with indicated antibodies. Antibody for CD47 (Cell Signaling, Cat. 36096), DNAJC13 (Thermofisher Scientific, Cat. 702773), GAPDH (Santa Cruz, Cat. sc-32233).

### Flow cytometry analysis

Cells were harvested, washed twice with ice-cold PBS containing 2% FBS (FACS buffer), and incubated with anti-mouse CD47-FITC antibody (BioLegend, Cat. 323108) for 30 minutes at 4°C in the dark. After staining, cells were washed twice with FACS buffer and resuspended in PBS for analysis. Flow cytometry was performed on a BD LSRFortessa (BD Biosciences), and data were analyzed using FlowJo software (v10). Live cells were gated based on forward and side scatter properties, and CD47 expression was quantified as mean fluorescence intensity (MFI). Unstained and isotype control samples were included in all experiments to set gates and background fluorescence.

### *In vitro* phagocytosis assay

0.5 × 10^5^ tumor cells labelled with CFSE (eBioscience, Cat. C34554A) were seeded overnight in 96-well cell culture plates. The next day, tumor cells were pre-incubated with different treatments for 1 h before the addition of 0.5 × 10^5^ macrophages labelled with Far Red (eBioscience, Cat. C345564A). The cells were co-cultured for 3 h at 37 °C with the different treatments in serum-free or serum-containing medium. The cells were then washed three times, collected and resuspended in PBS containing 1% BSA. Phagocytosis was analyzed with a CytoFLEX flow cytometer (Beckman Coulter) and calculated as the percentage of CFSE+ cells within the Far Red labelled macrophages.

### RNA isolation and RT-qPCR

Total RNA was prepared using TRIzol (Invitrogen) and reverse-transcribed using the PrimeScript RT Reagent Kit with the genomic DNA Eraser (Takara, RR047A). The qPCR reactions were run in an ABI Q6 RT-PCR instrument. Levels of DHPS mRNA were detected by the TaqMan MicroRNA assay (ABI Scientific) and normalized by β-actin mRNA. The primers used in this study were as follows:

β-Actin forward: CACCATTGGCAATGAGCGGTTC.

β-Actin reverse: AGGTCTTTGCGGATGTCCACGT.

DNAJC13 forward: CTTGTGGCACAGTCTGTGAA.

DNAJC13 reverse: TGATGGCAGGTTCTTTCCAG.

CD47 forward: AGCAGGGTCAGTTTGATGCAG.

CD47 reverse: TCCGTTTCTGGTAGCCTGTG.

### IHC staining and scoring

Slides deparaffinization, antigen retrieving, and blocking were performed according to protocols describle (33). Anti-CD47 antibody was from CST (Cell Signaling, Cat. 36096), anti-Ki-67 antibody was from Abcam (Abcam, Cat. ab16667) and anti-cleaved caspase 3 was from was Cell Signaling Technology (Cell Signaling, Cat. 9664S). Cell nuclei were stained with hematoxylin. Ventana iScan HT (Roche) was used for slide scanning with a ×20 objective lens.

### Animal model

For *in vivo* xenograft studies, 1 × 10^5^ B16F10A cells were subcutaneously injected into right flank of 6-week-old female C57BL/6J mice (The Jackson Laboratory, USA). Cells were suspended in phosphate-buffered saline (PBS) and mixed with Matrigel in 1:1 ratio (volume). Five mice were used per group. Mice with palpable tumors of similar size on day 5 were treated with anti-CD47 antibody (Bio X Cell, Cat. BE0270) *in vivo*. 100ug mice anti-CD47 antibody was given intraperitoneal every other day for two weeks. The mice were anesthetized using 2-3% isoflurane delivered in oxygen at a flow rate of 1.0-1.5 L/min and positioned on a platform with a cerrobend jig shielding the body. Tumor sizes were measured by an electronic caliper twice a week starting at 6 days post injection. And the tumor volume (V) was calculated by the formula: V = 1/2 × length × width^2^. Mice were euthanized by CO2 inhalation (50-60% of the chamber volume per minute) at the end of the experiment and the tumors were excised for subsequent analysis. The animals were housed in pathogen-free facilities. All animal experiments were approved by the MD Anderson Cancer Center Animal Care and Use Committee.

### Bioinformatics analyses

#### Co-expression analysis

Gene co-expression analysis was performed using publicly available datasets from the Cancer Cell Line Encyclopedia (CCLE) and The Cancer Genome Atlas (TCGA). For CCLE, normalized RNA-seq expression data were downloaded from the DepMap portal (https://depmap.org/portal/), and Pearson correlation coefficients were calculated to assess the relationship between DNAJC13 and CD47 expression across various cancer cell lines, including breast cancer, melanoma, and colon cancer. For TCGA, gene expression correlations were evaluated using the GEPIA2 web server (http://gepia2.cancer-pku.cn/), which integrates TCGA and GTEx RNA-seq data processed by a unified pipeline. Only tumor samples were included in correlation analyses, and results were visualized using scatter plots with fitted regression lines. Statistical significance was assessed by calculating Pearson’s correlation coefficient (R) and p-values provided by GEPIA2.

#### Immune infiltration analysis

The relationship between gene expression and immune cell infiltration was analyzed using the TIMER2 web application (http://timer.cistrome.org/). TCGA pan-cancer RNA-seq data were used to evaluate correlations between DNAJC13 or CD47 expression and the estimated abundance of infiltrating macrophages (total, M1, and M2 subsets where available). TIMER2 employs multiple deconvolution algorithms, including CIBERSORT, EPIC, and quanTIseq, to estimate immune cell infiltration levels based on bulk RNA-seq data. Correlation analyses were performed using the “Gene” module of TIMER2, which calculates Spearman correlation coefficients while adjusting for tumor purity. Correlation plots were generated directly from TIMER2, and only correlations with p < 0.05 were considered statistically significant.

#### Survival analysis

The prognostic relevance of DNAJC13 expression was assessed using TCGA patient survival data. GEPIA2 was used to perform Kaplan–Meier survival analyses for overall survival (OS) and disease-free survival (DFS) across multiple cancer types. Patients were stratified into high and low DNAJC13 expression groups using the median expression as a cutoff. Hazard ratios (HR) and 95% confidence intervals (CI) were calculated using a Cox proportional hazards model, and statistical significance was determined by the log-rank test. Survival curves were generated by GEPIA2 and further annotated for clarity.

### Statistical analysis

Data analyses were performed with unpaired t test or one-way analysis of variance (ANOVA) using GraphPad Prism software (version 8.0), unless otherwise noted. A P value <0.05 was considered statistically significant.

## Data Availability

The original contributions presented in the study are publicly available. This data can be found here: https://www.ncbi.nlm.nih.gov/geo/query/acc.cgi?acc=GSE315389.

## Supplementary material

Supplementary Figure 1 Quality control of CRISPR screen. **(A)** Deep-sequencing reads distribution in B16F10A, MC38 and EMT6 cells. The distributions of each sgRNA are presented. **(B)** CD47 and DNAJC13 sgRNA reads in these CRISPR screens. **(C)** Go enrichment analysis of significant negative regulators of CD47 expression (|NormZ| > 3) in EMT6 cells.

Supplementary Figure 2 Positive correlation between DNAJC13 and CD47 expression in cancer cell lines. Scatter plots show the correlation between DNAJC13 and CD47 expression (log2 TPM) in breast cancer cell lines (left), colon cancer cell lines (middle), and melanoma cell lines (right) derived from the Cancer Cell Line Encyclopedia (CCLE) dataset. The red line represents a fitted linear regression, and the shaded area indicates the 95% confidence interval, demonstrating a positive association between DNAJC13 and CD47 expression across all three cancer types.

Supplementary Figure 3 DNAJC13 KO and its impact on tumor growth *in vivo*. **(A)** Quantitative RT-PCR analysis showing significant reduction in CD47 mRNA expression in CD47-KO cells compared to parental controls in B16F10A, MC38, and EMT6 cell lines. Results are representative of at least two independent experiments. **(B)** Flow cytometry analysis confirming loss of CD47 surface expression in CD47-KO cells. Data represent mean fluorescence intensity (MFI) of CD47 staining in B16F10A, MC38, and EMT6 cell lines. Results are representative of at least two independent experiments. **(C)** Quantification of phagocytosis in DNAJC13-deficient and control cells. Statistical analysis was performed using at least three independent experiments, and significance is indicated in red in the bar graphs. Data are presented as mean ± SEM. DNAJC13 knockout significantly increased macrophage-mediated phagocytosis compared with control cells. **(D)** Briefly, 1×10^5^ cancer cells (mixed with Matrigel in 1:1 ratio) were subcutaneously injected into the right flank of 6-week-old female BALB/cJ or C57/B6 mice. Mice were randomly assigned to four groups (n=5). The tumor volume (V) was calculated by the formula V = 1/2 × length × width^2^. Tumor growth curves of different treatment groups are presented. The tumor volumes shown at each point where the average from 5 tumors.
