# Supplementary figures and images for: FACS-based genome-wide CRISPR screening platform identifies modulators of CD47
Source: Front Immunol. 2026 Jan 12;16:1684539. doi: 10.3389/fimmu.2025.1684539 (PMC12832931; doi:10.3389/fimmu.2025.1684539)

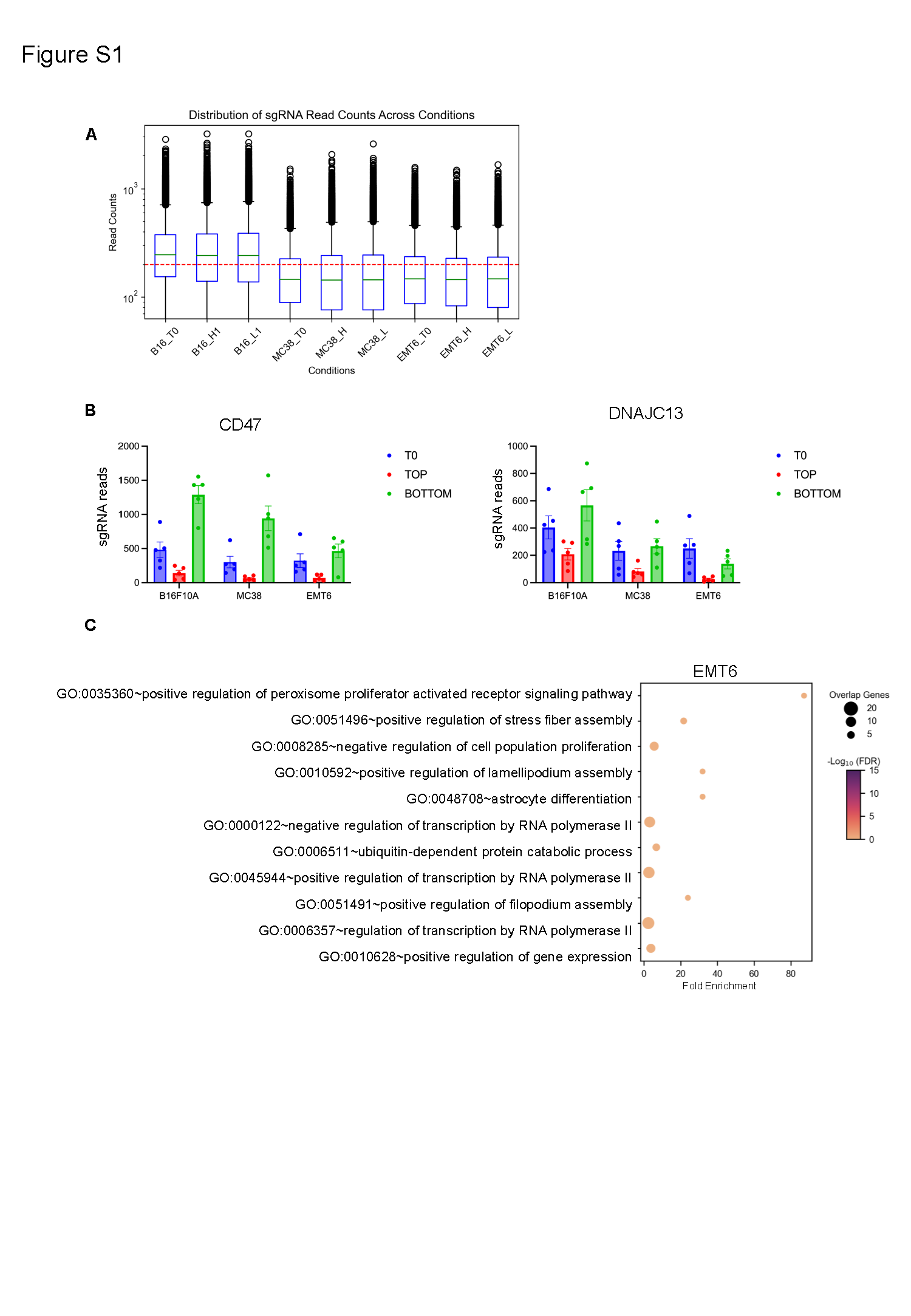

Supplement: Supplementary Figure 1 — Quality control of CRISPR screen. (A) Deep-sequencing reads distribution in B16F10A, MC38 and EMT6 cells. The distributions of each sgRNA are presented. (B) CD47 and DNAJC13 sgRNA reads in these CRISPR screens. (C) Go enrichment analysis of significant negative regulators of CD47 expression (|NormZ| > 3) in EMT6 cells. [file Image1.tiff]

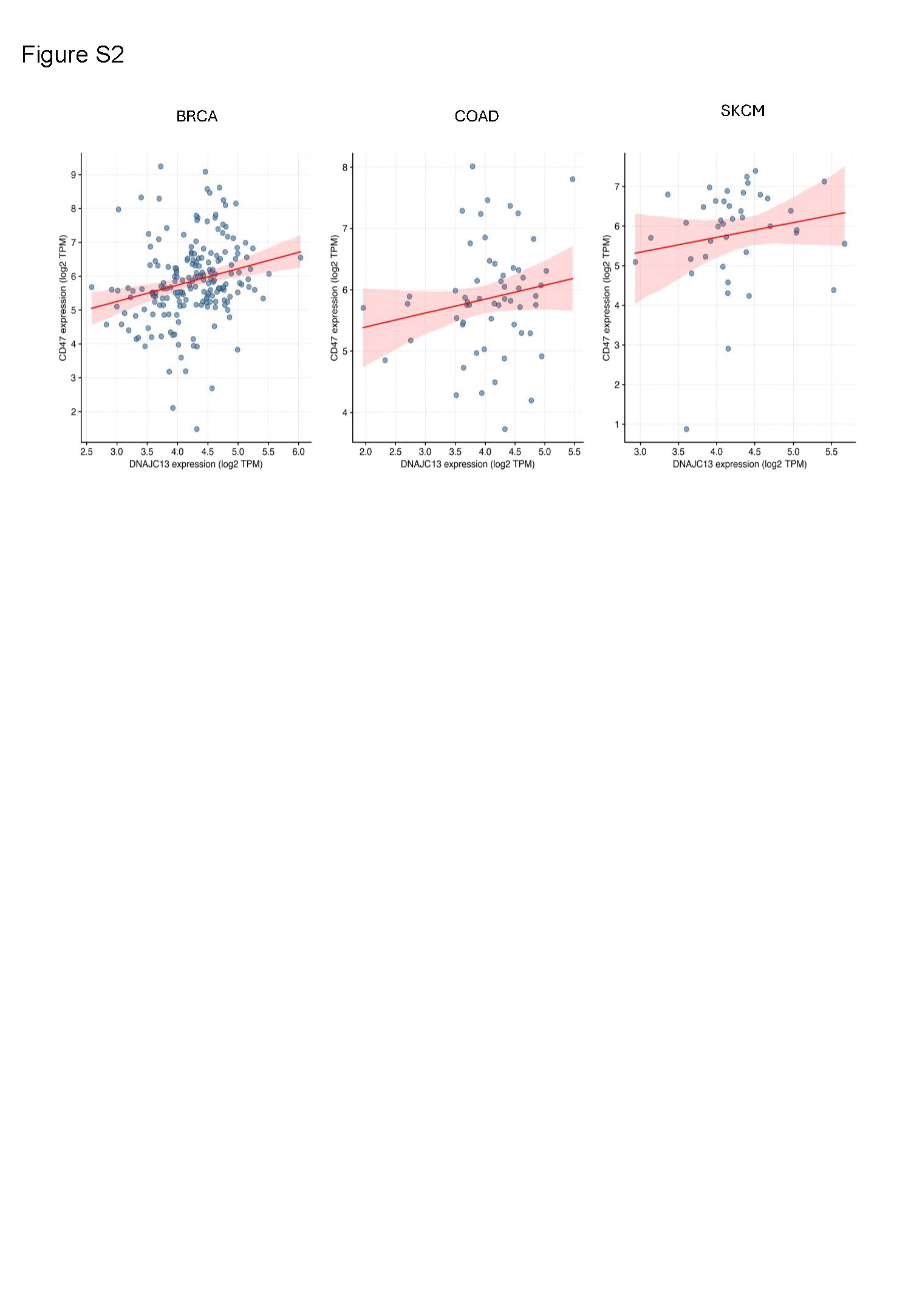

Supplement: Supplementary Figure 2 — Positive correlation between DNAJC13 and CD47 expression in cancer cell lines. Scatter plots show the correlation between DNAJC13 and CD47 expression (log2 TPM) in breast cancer cell lines (left), colon cancer cell lines (middle), and melanoma cell lines (right) derived from the Cancer Cell Line Encyclopedia (CCLE) dataset. The red line represents a fitted linear regression, and the shaded area indicates the 95% confidence interval, demonstrating a positive association between DNAJC13 and CD47 expression across all three cancer types. [file Image2.tiff]

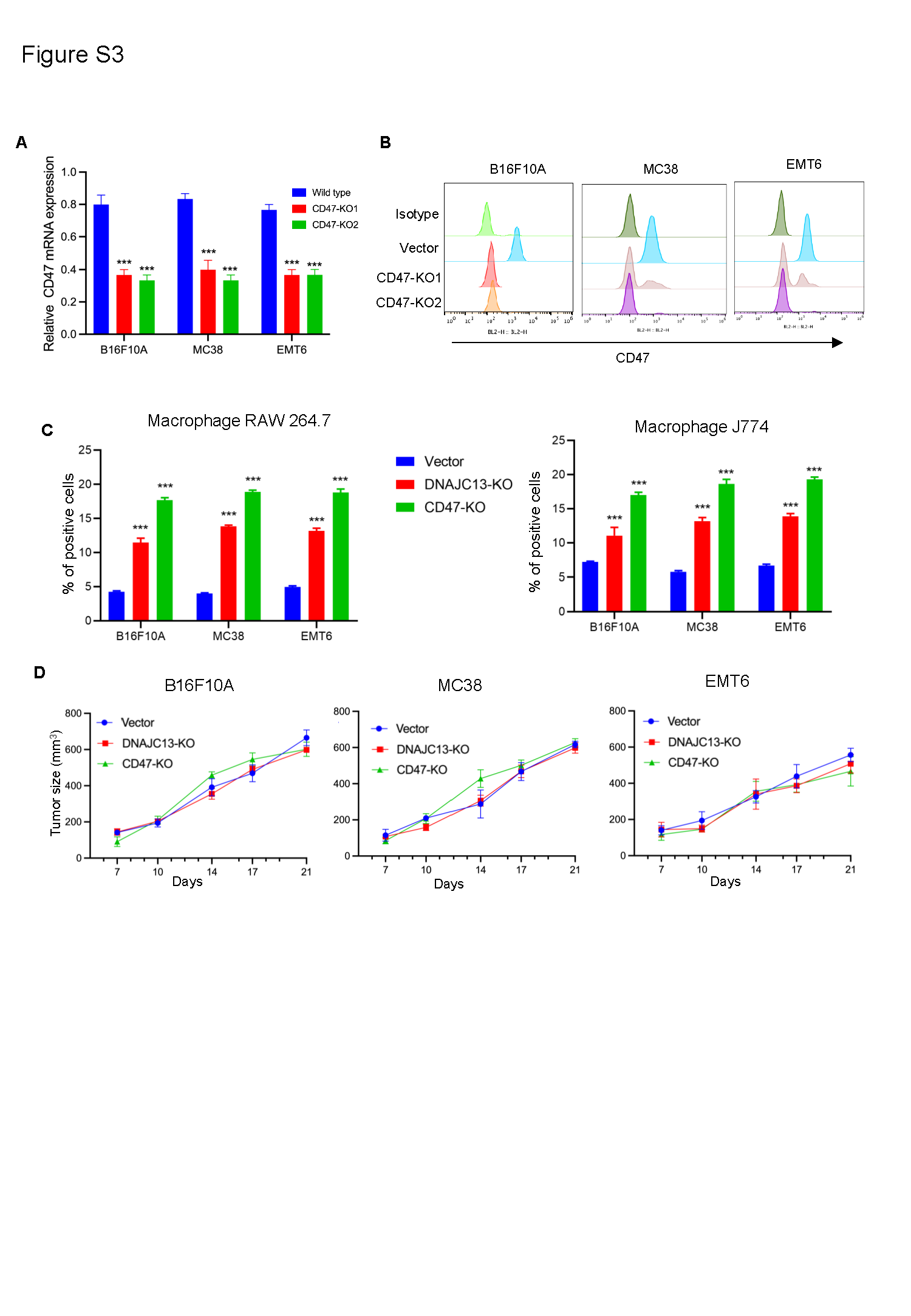

Supplement: Supplementary Figure 3 — DNAJC13 KO and its impact on tumor growth in vivo. (A) Quantitative RT-PCR analysis showing significant reduction in CD47 mRNA expression in CD47-KO cells compared to parental controls in B16F10A, MC38, and EMT6 cell lines. Results are representative of at least two independent experiments. (B) Flow cytometry analysis confirming loss of CD47 surface expression in CD47-KO cells. Data represent mean fluorescence intensity (MFI) of CD47 staining in B16F10A, MC38, and EMT6 cell lines. Results are representative of at least two independent experiments. (C) Quantification of phagocytosis in DNAJC13-deficient and control cells. Statistical analysis was performed using at least three independent experiments, and significance is indicated in red in the bar graphs. Data are presented as mean ± SEM. DNAJC13 knockout significantly increased macrophage-mediated phagocytosis compared with control cells. (D) Briefly, 1×10^5^ cancer cells (mixed with Matrigel in 1:1 ratio) were subcutaneously injected into the right flank of 6-week-old female BALB/cJ or C57/B6 mice. Mice were randomly assigned to four groups (n=5). The tumor volume (V) was calculated by the formula V = 1/2 × length × width2. Tumor growth curves of different treatment groups are presented. The tumor volumes shown at each point where the average from 5 tumors. [file Image3.tiff]
